# Use of YouTube as a Source of Information for Quitting or Cutting Down Alcohol

**DOI:** 10.3389/fpubh.2021.787994

**Published:** 2021-12-17

**Authors:** Abdullah Al Mahmud, Anh Le, Omar Mubin

**Affiliations:** ^1^Centre for Design Innovation, Swinburne University of Technology, Hawthorn, VIC, Australia; ^2^School of Computer, Data and Mathematical Sciences, Western Sydney University, Penrith, NSW, Australia

**Keywords:** YouTube, alcohol cessation, content analysis, alcohol, social media, human-computer interaction

## Abstract

**Background:** Although research has been done on considering YouTube for dissuading and encouraging unhealthy behaviours such as smoking, less focus has been placed on its role in quitting or cutting down alcohol. This study aims to analyse the alcohol cessation videos available and accessible on YouTube to gain a more in-depth insight into the ways that YouTube as a platform is being used to persuade with relation to alcohol cessation.

**Methods:** We systematically searched for content on YouTube related to alcohol cessation and these videos were analysed and evaluated for the format, themes, specific alcohol cessation advice, and uploader.

**Results:** The results demonstrated that the collected alcohol cessation videos included a fairly even presence of the themes of discussing the negative impacts of alcohol and the benefits of quitting or staying away from it. At the same time, however, we found the videos were not sourced from professional institutions, such as government or anti-alcohol misuse non-government organisations.

**Conclusion:** More research is needed to investigate utilising YouTube to support those looking to quit or cut down alcohol.

## Introduction

Drinking-related incidents and illnesses are on the rise all over the world (1). Social problems such as violence, family breakdown and child abuse and neglect as the consequences of alcohol misuse remain a serious problem (1). According to Ritchie and Roser (2), people older than 15 drink 53 bottles of alcohol per year and it indicates how alcohol use is a major risk factor for significant health damage worldwide (3).

Efforts must continue to be made in dissuading unhealthy levels of drinking and encouraging people with alcohol use disorder (AUD) to quit. As far as unexplored avenues are concerned, social media platforms such as Facebook, Twitter, and YouTube have already been acknowledged as wielding great influence (4). YouTube remains one of the most popular video-sharing platforms (5). There is empirical evidence that suggests that YouTube can indeed influence and shape the perspectives of its users (6). There is already evidence to suggest that even in a self-help format, un-guided by human interaction; technology can be used to some degree to deliver meaningful interventions into unhealthy lifestyle behaviours, even specifically alcohol addiction (7, 8). It can be reasoned then that even in a setting like YouTube where interaction is limited to the user comments section, a self-help system may provide meaningful support to those who struggle with alcohol use. There is also substantial information to suggest that YouTube can indeed be used as a viable and effective means of providing health information (9).

YouTube can be used as a means of disseminating pro-alcohol messages. Cranwell et al. (10) found that with a “ten-second interval content analysis” of the most popular music videos on YouTube in the United Kingdom, watched by many British teenagers, “particularly girls,” 22% of all videos presented tobacco imagery, whilst 45% presented alcohol branding or imagery. Barry et al. (11) suggested that even if alcohol manufacturers and stakeholders in the alcohol industry were not actively attempting to utilise YouTube as a means of propagating pro-alcohol messages, at the very least, they were not seemingly developing any self-imposed rules to avoid advertising to adolescents.

As a platform, YouTube shows great promise and might be harnessed as an effective method of persuading against alcohol misuse. However, to the best of our knowledge, literature concerning alcohol cessation messages on YouTube is lacking. Therefore, the aim of the study is to analyse and categorise the types of alcohol cessation videos available and accessible on the YouTube to gain a more in-depth insight into the ways that YouTube as a platform is being used to persuade with relation to alcohol cessation.

## Methods

The methodology used in this study is based on the methodology of a previous study (12) on the information available on YouTube for smokers with mental illnesses, and that evaluated the salience of each video.

### Phase One – Video Selection

As a preliminary investigation into YouTube as a platform for disseminating alcohol cessation messages and persuading people to stop drinking irresponsibly, one of the key factors in our research is not just the videos most salient to individuals who both utilise YouTube, but individuals who are on top of that looking to stop drinking alcohol. To do that, a systematic search of YouTube for anti-alcohol, or more specifically, alcohol cessation-related videos, was performed by searching using two different keyword phrases.

The key search phrase “How to stop drinking on your own” was first searched end of 2017 and early 2018, using the YouTube website's in-built search results filter ‘Filter: Sort By Relevance' and the first 20 videos, or more accurately the first page of results links were considered. The search by “relevance” was chosen first due to its nature as the default search filter option on the YouTube website, and thus the search algorithm most users were most likely to use.

Following that, the same search phrase “How to stop drinking on your own” was then filtered with “Filter: Sort By View Count” and again, the first page of results links were collected. The rationale behind the search by “view count” was the desire to find the most popular and viewed videos behind the key search words. The above two steps were repeated for the search phrase “How to quit alcohol” to provide a complete list, producing a list of (2 × 2 × 20) 80 videos.

**Figure 1:**
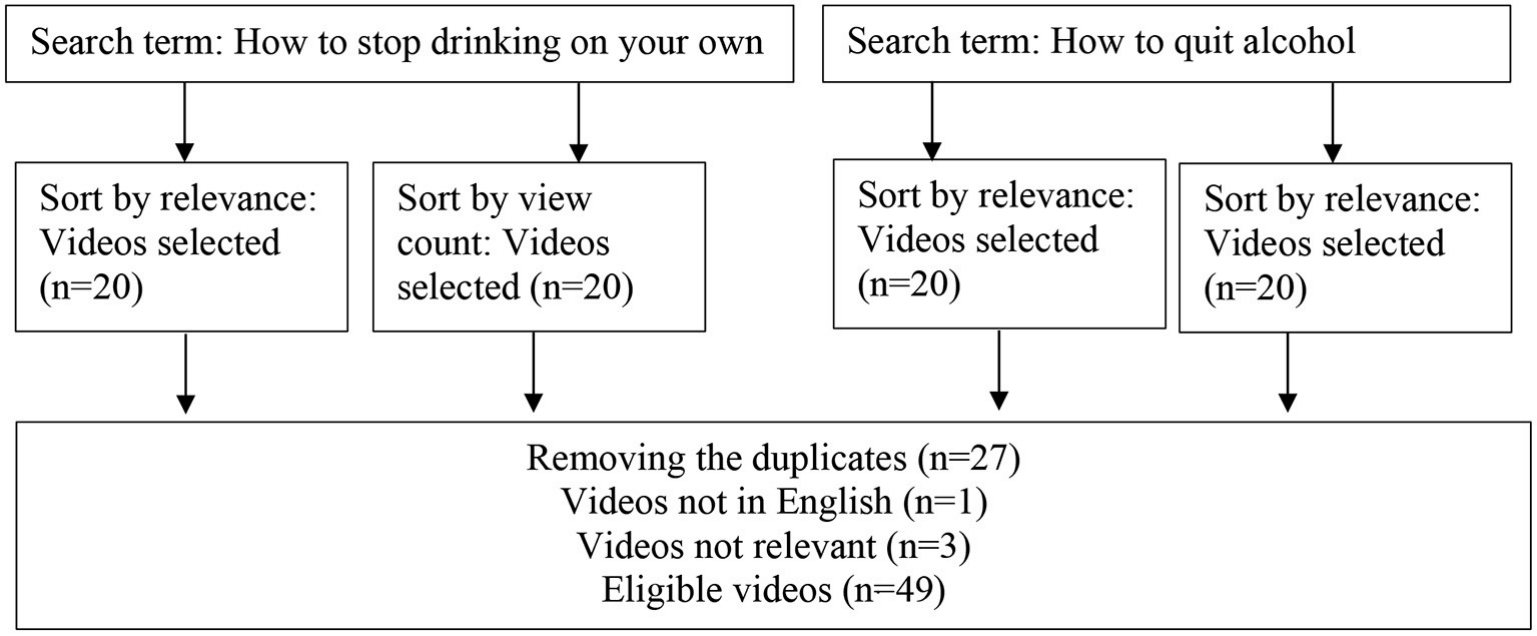
Flowchart for selecting the eligible videos.

The rationale behind the first 20 videos being taken was based in various independent market research studies that provided strong evidence that over 90% of all users would only consider the first page of results (13). Also, the number 20 is the default setting on YouTube, with regards to the number of videos displayed on the first page. This list of 80 YouTube video links was then filtered once more to remove duplicate results from the video pool, reducing the number of video links to 53, and the view-counts of each video linked were then taken on the within 10 min for posterity and possible comparison. The result was a list of 53 video links collated with the name and search method, ready for further screening (see Figure 1 for flow chart depicting search results).

### Phase Two – Analysing Videos and Establishing Rigour

The second step of the study involved categorising the videos. Due to the lack of similar studies in the past, there were no criteria evident in the context of the YouTube platform for specifically alcohol cessation-related videos. For this reason, a preliminary video content coding scheme was devised (Table 1), based on Sharma et al. (12) study. The video content coding scheme comprised of five questions, roughly encompassing the format of the video, the channel or uploader of the video, the themes present in the video, the presence or absence of certain elements of advice, and the presence or absence of specific types of people in the video.

**Table 1:** Video contents coding scheme.

**Questions**	**Possible answers**
1. How would you best describe the video?	• Help from a professional (A) • Advice from a peer and/or personal experiences (B) • Structured progress diary (C) • Documentary (D) • Informative presentation (E) • Newsclip (F) • Other (G)
2. How would you best categorise the video uploader?	• Peers/fellow with AUD (A) • Registered professional (B) • Health-related YouTube channel (C) • NGO (D) • GOV (E) • Blogger/Other Youtuber (F)
3. What kinds of themes were present in the video?	• Discussing the impact of drinking (A) • Discussing the benefits of quitting (B) • Advice on how to quit alcohol, and stay away (C) • Advice on possible problems and setbacks during the process (D) • Entertainment (E) • Other (F)
4. Did they suggest any of the following advice? Y/N	• Seeking advice from the health care provider • Seeking advice from community-based help groups • Simply cutting down
5. Did they feature any of the following in the video? Y/N	• Registered medical professional • Recovering person with AUD (pubescent) • Recovering person with AUD (young adult) • Recovering person with AUD (other) • Victim of an alcohol-related incident

After thoroughly watching each video, the video contents coding scheme was consulted, and the video was analysed and categorised as appropriately as possible (see Supplementary Data for the links to the videos). A video was coded under the category “Help from a professional” if it provided professional or organisational help to quit or cut down on alcohol. If a video provided any factual information to quit or cut down on alcohol (such as related to statistics), it was coded under the category called 'Informative presentation'.

For the multiple-choice questions 1 and 2, the “Other” option was made available to avoid unconscious bias in rationalising the inclusion of a video in more specific choices. The “Other” choice was also included for the function of providing a warning sign that the video contents coding scheme was ill-suited to the actual available YouTube video content. Thus, a large number of “Other” results would act as an indicator that the choice options would be required to be rechosen to better categorise the videos either in a repeat of this study or in future studies. For this reason, multiple-answer question 3 was also made to include and the “Other” option. Questions 4 and 5 were designed to flag the occurrence of points of interest. Due to the mutually exclusive nature of the choices presented in Questions 3, 4 and 5, each possible category was recorded as a binary “1” or “0,” where “1” represents the category's presence in the video, and “0” illustrates the category's absence.

Due to the possibility of subjectivity and invalidity, a second researcher was tasked with categorising a randomly taken 10% of the videos from the total sample pool. The analysis and categorisation performed by the second researcher was done completely independently, with no influence or input from the first researcher. The results were then compared to test the interpretations of the categories in the video contents coding scheme, and any differences in answers and categorisation were closely examined. Disagreements were discussed thoroughly among the researchers before agreeing on a specific category. Once the categorisation and evaluation of the 49 videos was completed, results were tallied for the purpose of analysing any possible trends.

## Results

### Video Search Results

Each of the 53 videos on the results list provided in the first phase of the study was then watched from the beginning of the video until the end. Videos that were entirely irrelevant to alcohol cessation, as well as videos not in the English language, however, were disqualified from the analysis. In total, four videos were disqualified, leaving a total of 49 videos to categorise and analyse.

### Overview of the Videos

#### Video Formats

Of the different possible video formats, “Advice from a peer and/or personal experiences” was by far the most prevalent at 17 videos (see Table 2). Videos in the format of “presentations” were a not too distant second. In contrast, despite the prevalence of “advice from a peer and/or personal experiences” videos in the search results, the median view count for such videos was relatively low at 76,945, losing to “presentations” at 176,848 and “help from a professional” at 180,292.

**Table 2:** Video contents characteristics and view count.

**Content**	**Tally**		**Views**	
**Questions**	**No**.	**%**	**Total**	**Median**
**1. How would you best describe the video?**				
Help from a professional	7	14.29%	1,659,555	180,292
Advice from a peer and/or personal experiences	17	34.69%	3,503,710	76,945
Structured progress diary	2	4.08%	93,493	46,747
Documentary	1	2.04%	285,864	N/A
Informative presentation	12	24.49%	7,192,887	176,848
Newsclip	0	0.00%	N/A	N/A
Other	9	18.37%	11,981,419	104,022
**2. How would you best categorise the video uploader?**				
Peers/fellow person with AUD	1	2.04%	42,476	N/A
Registered professional	4	8.16%	381,425	64,313
Health-related YouTube channel	21	42.86%	3,442,362	80,034
NGO	1	2.04%	582,483	N/A
GOV	0	0.00%	N/A	N/A
Blogger/Other Youtuber	21	42.86%	20,268,182	276,801
**3. What kind of themes were present in the video?**				
Discussing the impact of drinking	18	36.73%	7,980,094	136,105
Discussing the benefits of quitting	21	42.86%	14,794,770	89,590
Advice on how to quit alcohol, and stay away	21	42.86%	3,129,083	85,177
Advice on possible problems and setbacks during the process	17	34.69%	2,157,997	76,945
Entertainment	9	18.37%	14,330,422	355,106
Other	3	6.12%	5,746,667	975,437
**4. Did they suggest any of the following advice? Y/N**				
Seeking help from health care provider	1	2.04%	63,668	N/A
Seeking help from community based help groups	3	6.12%	688,627	63,668
Simply cutting down	14	28.57%	1,805,325	87,384
**5. Did they feature any of the following in the video? Y/N**				
Registered medical professional	0	0.00%	N/A	N/A
Recovering person with AUD (pubescent)	0	0.00%	N/A	N/A
Recovering person with AUD (young adult)	0	0.00%	N/A	N/A
Recovering person with AUD (other)	21	42.86%	3,464,200	76,945
Victim of alcohol-related incident	0	0.00%	N/A	N/A

#### Types and Sources of Videos

Of the possible YouTube channels uploading these videos, over 80% of them comprised Health-related YouTube channels and other non-professional, non-government affiliated channels. Health-related YouTube channels uploaded 42.86% of all videos, whilst professionals of any sort only uploaded 8%, with no government presence at all.

Four-fifths of all the videos examined came from personal YouTube channels that were either general entertainment such as blogging, or personal YouTube health-related blogging channels. In comparison, there was only a single video uploaded by a professional Non-Government Organisation. Of the four videos uploaded by a professional, three of them were registered hypnotherapists while one of them was the professional motivation speaker, who previously misused alcohol, meaning that none of the videos from the sample pool considered emerged from medical professionals or professionals in fields more traditionally linked to helping with going sober.

Following pre-study expectations, the videos included a roughly even distribution of the themes of discussing the negative impacts of alcohol, discussing the benefits of quitting alcohol, giving advice on how to quit alcohol and stay sober, and finally advice on possible difficulties or setbacks during the struggle to stay sober and stay away from alcohol, at 36.73, 42.86, 42.86, and 34.69% of videos, respectively.

Fourteen of the 49 videos suggested simply cutting down or willpower as the primary, or at least an important method of abstaining from alcohol and staying sober, whilst only 3 videos suggested the idea of seeking advice or support from community-based help groups, and only 1 of the 49 videos suggested seeking help from a health care provider.

## Discussion and Conclusion

It becomes obvious from the results that most content producers for alcohol-cessation-related content are either health-related YouTube channels or simply everyday YouTube content producers. This suggests a distinct lack of professional presence on YouTube regarding supporting struggling people with AUD and those looking to quit. Similar results were observed in a study where researchers investigated smoking cessation videos on YouTube (14).

Furthermore, not a single government-produced video was present in the 49 videos selected; there were even videos uploaded by a person who previously misused alcohol and a casual user of YouTube. This suggests either a lack of professional motivation to utilise YouTube as a means of disseminating alcohol cessation support content or that such content from profession NGOs and the government do exist but are failing to gain views or turn up on YouTube's search algorithms. The seeming lack of professionally organised alcohol cessation support videos have significant implications for the current use of YouTube as a persuasive technology platform.

Examining the formats of the videos points to videos structured as advice on alcohol cessation from fellow people with AUD or people who previously misused alcohol emerged as a popular mode, which seemed a recommended strategy in a previous study (15). At the same time, however, the median view count is one of the lowest there, especially compared to the hypnotherapy videos, so this may suggest a trend toward YouTube's user preference for hypnotherapy as a means of combating alcoholism.

As was expected from before the categorisation, the videos included a reasonably even presence of the themes of discussing the negative impacts of alcohol, discussing the benefits of quitting alcohol, giving advice on how to quit alcohol and stay sober, and finally, advice on possible difficulties or setbacks during the struggle to stay sober and stay away from alcohol. This suggests that although the content most readily available may not be sourced from more professional institutions such as established NGOs or the government, content that may be helpful to struggling people with AUD is indeed still available. Therefore, it is apparent that existing YouTube videos may potentially support intervention for alcohol misuse though the video contents need to be sourced from legitimate sources to provide the optimal persuasion, as mentioned in Lehto and Oinas-Kukkonen (16).

Regarding specific advice, surprisingly few videos were noted that advised seeking help from health care providers or help from community-based support groups, with only 1 of the former, and 3 of the latter. In comparison, 14 videos, or almost 30% of the videos, including the message that the best method of quitting alcohol is simply to abstain or “want it.” Coupled with the noted popularity of the hypnotherapy videos, this may spell a trend of belief toward more non-traditional methods of alcohol cessation, as opposed to the traditional advice of seeking help from health care providers or group-based solutions such as Alcoholics Anonymous. It was noted that one video specifically stated these methods as being useless.

Finally, it was surprising to note the lack of discussion on more specific issues, such as teenage people with AUD, victims of alcohol related incidents, as well as the lack of registered medical professionals brought in to speak on these videos. However, considering what the study discovered about the lack of professionally organised alcohol cessation content creators, this may be expected due to the logistics in doing so.

There was a crucial lack of organised government or non-government organisation presence, suggesting either underutilisation of YouTube as a serious platform for anti-alcohol misuse outcomes, or a failure to produce engaging and relevant video content. Social media can assist governments in providing health information to citizens to manage various public health issues (17). Government organisations should utilise the power of YouTube videos to support people for quitting or cutting down on alcohol. The significant presence of government and non-government organisations will ensure that authentic and proven strategies are provided. Government organisations in some countries, for instance, see BetteHealtChannel (18), provide web-based information on the negative effects of alcohol. However, it would have been even more effective if some information would be disseminated through YouTube.

Very few videos suggested the use of healthcare organisations or going to a community-based help group for help in quitting alcohol, hinting at possible unpopularity. In contrast, there were several hypnotherapy-based alcohol cessation videos, suggesting an unexpected level of interest in hypnotherapy as a means of quitting and staying away from alcohol, which in turn suggests a possible avenue of approach for future anti-alcohol misuse campaigns.

There is no doubt that YouTube presents readily accessible anti-alcohol misuse, and alcohol cessation content. Perhaps due to its nature as a user-content sharing platform, the majority of the videos found were based around a format of having a person who previously misused alcohol talk to the audience in a video blog or “v-log” format. If these vlogs are streamed live on YouTube, viewers could also engage through live chat, which is an added functionality that a platform such as YouTube can provide. Moreover, YouTube videos can easily be integrated and embedded into numerous other social media platforms, which are just as if not more popular (Twitter, Instagram, and Facebook). This can lead to great proliferation, outreach and views to support the early intervention for alcohol misuse.

The study is limited to a sample size of only 49 videos to analyse. However, our study is to be considered as the first investigation into the landscape of alcohol cessation content on the YouTube platform. This study will allow for further investigations into the use of YouTube as a platform for persuasion for quitting or cutting down alcohol.

## Data Availability Statement

The original contributions presented in the study are included in the article/Supplementary Material, further inquiries can be directed to the corresponding author/s.

## Author Contributions

AAM and OM contributed to the conception, design of the study, and manuscript revision. AL collected the data with the assistance of AAM and OM. AL performed the initial data analysis and drafted the research report. AAM prepared the first draft of the manuscript. All authors contributed to the article and approved the submitted version.

## Conflict of Interest

The authors declare that the research was conducted in the absence of any commercial or financial relationships that could be construed as a potential conflict of interest.

## Publisher's Note

All claims expressed in this article are solely those of the authors and do not necessarily represent those of their affiliated organizations, or those of the publisher, the editors and the reviewers. Any product that may be evaluated in this article, or claim that may be made by its manufacturer, is not guaranteed or endorsed by the publisher.

## References

[1] National Health Medical Research Council. Alcohol Guidelines: Reducing the Health Risks. (2021). Available online at: https://www.nhmrc.gov.au/health-advice/alcohol (accessed September 30, 2021).

[2] Ritchie H Roser M. Alcohol Consumption (2018). Retrieved from: https://ourworldindata.org/alcohol-consumption

[3] Griswold MG Fullman N Hawley C Arian N Zimsen SR Tymeson HD. Alcohol use and burden for 195 countries and territories, 1990–2016: a systematic analysis for the Global Burden of Disease Study 2016. Lancet. (2018) 392:1015–35. 10.1016/S0140-6736(18)31310-2 30146330 PMC6148333

[4] Social Media Statistics Australia (2021). Available online at: http://www.socialmedianews.com.au/social-media-statistics-australia-january-2015/ (accessed January 21, 2021).

[5] Media Access Australia (2021). YouTube. Available online at: https://mediaaccess.org.au/web/social-media/youtube (accessed September 30, 2021).

[6] Rotman D Preece J. The'WeTube'in YouTube–creating an online community through video sharing. Int J Web Based Commun. (2010) 6:317–33. 10.1504/IJWBC.2010.03375529626045

[7] Riper H Kramer J Smit F Conijn B Schippers G Cuijpers P. Web-based self-help for problem drinkers: a pragmatic randomized trial. Addiction. (2008) 103:218–27. 10.1111/j.1360-0443.2007.02063.x 18199300

[8] Blankers M Koeter MW Schippers GM. Internet therapy versus internet self-help versus no treatment for problematic alcohol use: a randomised controlled trial. J Consult Clin Psychol. (2011) 79:330. 10.1037/a0023498 21534652

[9] Walton LR Seitz HH Ragsdale K. Strategic use of YouTube during a national public health crisis: the CDC's response to the 2009 H1N1 flu epidemic. Case Stud Strateg Commun. (2012) 1:25–37. Available online at: http://cssc.uscannenberg.org/wp-content/uploads/2013/10/v1art3.pdf

[10] Cranwell J Murray R Lewis S Leonardi-Bee J Dockrell M Britton J. Adolescents' exposure to tobacco and alcohol content in YouTube music videos. Addiction. (2015) 110:703–11. 10.1111/add.12835 25516167 PMC4402034

[11] Barry AE Johnson E Rabre A Darville G Donovan KM Efunbumi O. Underage access to online alcohol marketing content: a YouTube case study. Alcohol Alcohol. (2015) 50:89–94. 10.1093/alcalc/agu078 25411395

[12] Sharma R Lucas M Ford P Meurk C Gartner CE. YouTube as a source of quit smoking information for people living with mental illness. Tob Control. (2016) 25:634–7. 10.1136/tobaccocontrol-2015-052713 26758030

[13] Jacobson M. How Far Down the Search Engine Results Page Will Most People Go. (2017). Available online at: https://www.theleverageway.com/blog/how-far-down-the-search-engine-results-page-will-most-people-go/ (accessed November 28, 2021).

[14] Richardson CG Vettese L Sussman S Small SP Selby P. An investigation of smoking cessation video content on YouTube. Subst Use Misuse. (2011) 46:893–7. 10.3109/10826084.2011.570628 21599505

[15] Buscemi J Murphy JG Martens MP McDevitt-Murphy ME Dennhardt AA Skidmore JR. Help-seeking for alcohol-related problems in college students: correlates and preferred resources. Psychol Addict Behav. (2010) 24:571. 10.1037/a0021122 21198220 PMC4912043

[16] Lehto T Oinas-Kukkonen H. Persuasive features in web-based alcohol and smoking interventions: a systematic review of the literature. J Med Internet Res. (2011) 13:e1559. 10.2196/jmir.1559 21795238 PMC3222186

[17] Khan GF. Social Media for Government. Singapore: Springer (2017). p. 7–21. 10.1007/978-981-10-2942-4

[18] BetteHealtChannel (2021). BetteHealthChannel. Available online at: https://www.betterhealth.vic.gov.au/health/healthyliving/how-alcohol-affects-your-body (accessed November 28, 2021).

